# Case in Which Six Drachms of Arsenic Were Taken

**Published:** 1856-01

**Authors:** Wm. Webb

**Affiliations:** St. Louis


### *Case in which Six Drachms of Arsenic were taken. Hydratid Sesquinoxide of Iron administered. Recovery.* By WM. WEBB, M. D., of St. Louis.

I was called about ten and a half o'clock, on Sunday night, Sept. 2d, to see Mrs. O., who had been taken suddenly ill. I learned from her that she had procured from a druggist a parcel of arsenic for the ostensible purpose of killing rats; that, owing to a difficulty with her husband, she determined to de-

*See report of Society's proceedings, Ed. Monthly Journal of Medical Science, April, 1855, p. 350.—*Eds. Prof. Simpson's Work.*

†Ephemer. Nat. Cur., Dec. ii., An. v., Obs. 223.

stroy herself. I repaired to the apothecary's and learned from him, that she had procured that evening, about *six drachms* of arsenic acid—a specimen from the same parcel examined since by a practical chemist, (Mr. Brown) is pronounced unadulterated. She states "that she had eaten nothing all day, except a small piece of meat at dinner, and that she took *all* the arsenic she got from the druggist in half a tumbler of water, while the family were at supper, about six o'clock, and that she did not vomit until eight, and only vomited two or three times before my visit; thinks that she did not get up any of the powder, only water, with a little bile.

Found her, four and a half hours after the administration of the poison, retching and vomiting bile snd mucus; pale, with a dull expression of countenance, inclined to be stupid; skin cold and clammy; restless; pulse feeble and quick—about 120; violent burning pain in the epigastrium; tongue considered redder than natural. While the antidote was being procured, I vomited her freely with salt and mustard, and copious draughts of hot water, and nothing like arsenic in powder was emitted. The officinal hydrated sesquioxide of iron was now near five hours after the administration of the poison given; at first diluted with equal parts of water, owing to the difficulty in getting her to take it in the pulpy state, though in a short time she took freely of the magma, in doses of a tablespoonful every ten minutes, she vomiting, I think, twice; once half an hour after the administration of the first dose, and then some considerable time afterwards.

I learned from the druggist that she took all he had, about a pound, and I obtained two ounces from another store. The administration of the antidote was continued without any other adjuvants, except occasionally mustard poultices, until about eight o'clock next morning. I saw her at seven o'clock, Monday morning; expression of countenance better; face flushed; skin hot; pulse 108, full; has had several very copious watery evacuations; eyelids very much swollen, and conjunctiva injected; numbness of the extremities; pain in the lumbar region; no urine; tongue quite red; complains of rawness of the throat; great thirst. Ordered mustard sinapisms over the stomach; sulphate of morphia one-eighth of a grain, occasionally; to drink copiously of iced elm water; to be kept quiet. Ten o'clock.—Symptoms as before; no urine or stool. Six o'clock.—Fever; pulse full, 120; tenderness over the stomach and abdomen, very marked, though no undue heat of skin: feet numb, and occasionally severe pain running up the legs to the hip. Ordered the elm water and ice to be taken *ad libitum*, and to be cupped to twelve ounces over the epigastrium and abdomen; to take the morphia to relieve pain.

Tuesday morning—Did not get the cups; took the morphine only twice; much better; has slept well; urine about a pint, natural; no stool; wishes to take some toast, which I forbid; much less tenderness over the stomach and bowels; tongue better; throat still raw; wants to sit up. Ordered her to take nothing but mucilage, and keep quiet.

Wednesday.—Much better. Ordered an ounce each of castor oil and aromatic syrup of rhubarb, of which she is to take a tablespoonful every two hours until it operates.

Thursday.—Has had two evacuations from her bowels; very profuse and very black; all her symptoms improved.

Saturday.—She is up and looks pretty well, feels well, but is dull and listless; has been out several times; still, has some pain and numbness in her legs; tongue has a white coat around the edges, with a red patch half an inch wide in the centre; great thirst, with an insatiable appetite; eye-lids considerably swollen in the morning. Has continued to improve, and is at present quite well.

*Remarks.*—The points of interest in this case are, the quantity of arsenic taken, the length of time after, before the administration of the antidote; and the certainty of the antidotal powers of the protoxide of iron. We must suppose, from the quantity taken, symptoms, length of time taken, and upon an empty stomach, that absorption of a considerable quantity must have taken place; and the point of vital import in this case is, that if the protoxide of iron did act as the antidote, we may not dispair in its administration, even after a sufficient time has elapsed for the absorption of some of the poison.

Did the protoxide of iron unite with the arsenious acid in the stomach, forming the insoluble subarsenite of the protoxide of iron? or was the antidotal power exhibited in the blood after absorption? or both?—*St. Louis Med. and Surg. Journal.*

